# Yeast Particle Encapsulation of Azole Fungicides for Enhanced Treatment of Azole-Resistant *Candida albicans*

**DOI:** 10.3390/jfb15080203

**Published:** 2024-07-23

**Authors:** Ernesto R. Soto, Florentina Rus, Gary R. Ostroff

**Affiliations:** Program in Molecular Medicine, University of Massachusetts Medical School, Worcester, MA 01605, USA; ernesto.soto-villatoro@umassmed.edu (E.R.S.); florentina.rus@umassmed.edu (F.R.)

**Keywords:** yeast particles, glucan lipid particles, azole fungicides, drug resistance, terpenes, microencapsulation, biocide, controlled delivery

## Abstract

Addressing the growing problem of antifungal resistance in medicine and agriculture requires the development of new drugs and strategies to preserve the efficacy of existing fungicides. One approach is to utilize delivery technologies. Yeast particles (YPs) are 3–5 µm porous, hollow microspheres, a byproduct of food-grade *Saccharomyces cerevisiae* yeast extract manufacturing processes and an efficient and flexible drug delivery platform. Here, we report the use of YPs for encapsulation of tetraconazole (TET) and prothioconazole (PRO) with high payload capacity and stability. The YP PRO samples were active against both sensitive and azole-resistant strains of *Candida albicans*. The higher efficacy of YP PRO versus free PRO is due to interactions between PRO and saponifiable lipids in the YPs. Encapsulation of PRO in glucan lipid particles (GLPs), a highly purified form of YPs that do not contain saponifiable lipids, did not result in enhanced PRO activity. We evaluated the co-encapsulation of PRO with a mixture of the terpenes: geraniol, eugenol, and thymol. Samples co-encapsulating PRO and terpenes in YPs or GLPs were active on both sensitive and azole-resistant *C. albicans*. These approaches could lead to the development of more effective drug combinations co-encapsulated in YPs for agricultural or GLPs for pharmaceutical applications.

## 1. Introduction

Antifungal drug resistance poses great challenges to agriculture, as well as veterinary and public health [1,2,3,4,5,6,7]. Species belonging to *Candida*, *Aspergillus*, *Pneumocystis* and *Cryptococcus* are among the most common fungal pathogens exhibiting drug resistance [4,5,8]. There are four classes of antifungals used to manage and prevent fungal infections: (1) polyenes, which bind ergosterol and produce membrane pores, (2) echinocandins that inhibit the synthesis of β-1,3-glucan, (3) pyrimidine analogs, which inhibit synthesis of DNA and RNA, and (4) azoles that function by inhibiting sterol 14 demethylase and reducing ergosterol levels in the plasma membrane [9]. Of these groups, azoles are the primary class of fungicides for the prevention and effective treatment of fungal diseases in plants, animals, and humans, and they are also widely used in cosmetics and material preservation [3,10]. The extensive use of azole fungicides across different fields is due to the broad-spectrum range of fungal targets and relatively low cost. Unfortunately, the widespread use of azole fungicides and the similarities in chemical structure and mechanism of action of agricultural and medical azoles increases the emergence of resistant pathogenic strains with potential crossover of resistant strains from environment to clinical settings [2,3].

Addressing the challenges of antifungal drug resistance requires efforts to optimize sustainable agricultural practices that minimize the application of antifungals, mitigation of antifungal leakage to the environment, development of novel antifungal agents, and strategies of drug repurposing and combination therapies [2,5,11]. Given the prominent role of the azole class of fungicides for treatment of fungal diseases in many fields, one main research focus should be to preserve this class of antifungals for continued use in all areas [2,5].

The strategies to reverse resistance to azole fungicides can include combination therapies with compounds that either elicit antifungal effects or increase the pathogen susceptibility to the original antifungal drug. In this context, naturally occurring compounds are a primary source of bioactive materials with the potential to be used as chemosensitizers in combination therapies with existing antifungals [12]. For example, natural lignin was shown to reverse resistance to fluconazole in *C. albicans* though alteration of the glycolytic pathway [13], and terpenoids, the naturally occurring compounds that constitute the primary component of essential oils, can enhance the activity of fluconazole and other antifungal drugs against *Candida* strains [11,14]. The possibility of reversing antifungal resistance can provide the opportunity to resurrect the efficacy of existing, cost-effective drugs.

Yeast particles (YPs) are 3–5 µm porous, hollow particles derived from Baker’s yeast (*Saccharomyces cerevisiae*). We have developed the use of YPs as an efficient and flexible platform for the encapsulation of a wide range of small molecules and macromolecules within the hollow cavity of the particles for drug delivery and agricultural applications [15,16,17,18,19,20,21,22,23,24,25,26]. In this article, we present the results of the encapsulation of the agricultural azole fungicides tetraconazole (TET) and prothioconazole (PRO). These compounds were encapsulated in YPs with high efficiency (>90%) and high payload loading capacity (1:1 *w*/*w*). The YP-encapsulated PRO samples (YP PRO) were active against both sensitive and azole-resistant strains of *C. albicans.* The higher efficacy of YP PRO compared to unencapsulated PRO is due to the synergy between PRO and saponifiable lipids co-encapsulated in the YPs. These YPs are suitable for agricultural applications but cannot be used in most parenteral pharmaceutical applications due to their potential immunogenic effects. A highly purified form of YPs, glucan lipid particles (GLPs), were used to encapsulate PRO. The GLPs lack the saponifiable lipids of YPs and samples of GLP PRO are not active against azole-resistant *C. albicans.* The synergy of co-encapsulating a mixture of terpenes (geraniol, eugenol, thymol, GET 2:1:2 weight ratio) and PRO in both GLP and YP were evaluated, resulting in both GLP and YP samples being active against sensitive and azole-resistant *C. albicans.* The development of these yeast-particle-encapsulated fungicides has potential applications to target antifungal resistance in agriculture (YP fungicides) and pharmaceutical (GLP fungicides) applications.

## 2. Materials and Methods

Yeast particles (YPs) were purchased from Biorigin (Louisville, KY, USA). Technical-grade prothioconazole and tetraconazole were supplied from Sipcam Oxon (Lodi, Italy) and Eden Research plc (Oxfordshire, England), respectively. Terpenes (eugenol, thymol, and geraniol) were procured from Penta Manufacturing (Livingston, NJ, USA). All other reagents and solvents were obtained from Fisher Scientific (Waltham, MA, USA) or Sigma Aldrich (St. Louis, MO, USA). Yeast peptone dextrose (YPD) was prepared from Difco^TM^ yeast extract, Difco^TM^ Bacto peptone and dextrose (all materials obtained from Fisher Scientific) at a composition of 1% yeast extract, 2% peptone and 2% dextrose *w*/*v*.

### 2.1. Synthesis of Glucan Lipid Particles (GLPs)

The glucan lipid particles were prepared from YPs following the method previously reported for glucan particles (GPs) [15] but without the washing steps with organic solvents to avoid removal of non-saponifiable lipids. Yeast particles (100 g) are suspended in 1 L of 1M NaOH and heated to 85 °C. The cell suspension is stirred vigorously for 1 h at this temperature. The insoluble material containing the yeast cell walls is recovered by centrifugation (14,000 rpm, 30′). The pellet is then suspended in 1 M NaOH, heated, and stirred vigorously for 1 h at 85 °C. The suspension is allowed to cool to room temperature (20–25 °C) and the extraction is continued for a further 16 h. The insoluble residue is recovered by centrifugation, suspended in 500 mL of water and the pH of the particle suspension is brought to pH 4.5 with HCl. The insoluble residue is recovered by centrifugation and washed three times with water. The resulting slurry is placed in glass trays and dried under reduced pressure to produce a fine light-yellow powder.

### 2.2. YP Loading of Tetraconazole (YP TET)

Dry YPs were mixed with water (1 µL water/mg YP) for 12–18 h at 4 °C to obtain a uniform hydrated YP sample. Then, TET was added at weight ratios of 1:1 or 3:1 TET:YP and incubated at room temperature (20–25 °C) for a minimum of 24 h to allow for complete TET loading.

### 2.3. YP or GLP Loading of Prothioconazole (YP PRO or GLP PRO)

Dry YPs or GLPs were mixed with water (0.5 µL water/mg YP) for 12–18 h at 4 °C to obtain a uniform hydrated YP or GLP sample. Then, PRO was absorbed into YPs or GLPs by swelling the particles with a solution of 400 mg PRO/mL in acetone (2.5 µL acetone solution/mg YP or GLP) and incubated at room temperature (20–25 °C) for a minimum of 24 h to allow for complete PRO loading. The sample was dried under reduced pressure to remove solvent. To maximize loading of PRO into the hollow cavity of the particles, the hydration and absorption steps were repeated with 0.5 µL water/mg YP and 2 µL acetone/mg YP.

### 2.4. YP or GLP Co-Encapsulation of Terpenes and Prothioconazole

Samples co-encapsulating a mixture of terpenes—geraniol, eugenol and thymol (2:1:2 geraniol:eugenol:thymol, GET212)—and PRO were prepared following the procedure described above for YP PRO using the GET212 mixture as solvent for PRO (0.5 µL water/mg YP, 1.1 mg GET212/mg YP, 0.055 mg PRO/mg GLP or YP). 

### 2.5. Characterization of Payload Loading Efficiency

Samples of YP TET and YP PRO were stained with Nile red to qualitatively assess loading inside the hollow cavity of the particles by fluorescence microscopy of the encapsulated fluorescent fungicide-Nile red complex. Microscopy images were collected with an Olympus BX60 upright compound fluorescent microscope (ex 550 nm/em 570 nm, Olympus, Tokyo, Japan). Samples of YP- or GLP-encapsulated payloads (5–10 mg YP or GLP) were weighed, suspended in 1 mL of water, centrifuged at low speed (3000 rpm for 5 min) and the supernatant was collected. The pellets were extracted with a mixture of 10% water and 90% methanol (PRO and GET) or 10% water and 90% acetone (TET samples). The azole fungicides and terpenes were quantified in both supernatant (unencapsulated payload) and extracted pellets (encapsulated) by HPLC operated with 32 Karat^TM^ software version 7.0 (Beckman Coulter, Inc., Brea, CA, USA), using a Waters Symmetry^®^ C18 column (3.5 µm, 4.6 × 150 mm), flow rate of 0.8 mL/min, injection volume of 10 µL, detection by absorbance at 210 nm. The isocratic mobile phase conditions were acetonitrile:water 70:30 for quantification of samples containing only TET or PRO, and acetonitrile:water 60:30 for mixtures containing PRO and terpenes. The quantification of each payload was performed by measuring the peak area and interpolating the concentration using a calibration curve.

### 2.6. Payload Release from YPs or GLPs

Samples were suspended in water at a concentration of 10 mg PRO or TET/mL, and a set of samples were prepared by 10-fold serial dilutions down to a concentration of 0.01 mg PRO or TET/mL. The samples were incubated at 23 °C and aliquots were collected at predetermined times, centrifuged and the supernatant collected to measure payload released from the particles by HPLC.

### 2.7. Antifungal Activity Assays against Sensitive and Azole-Resistant Candida albicans Strains

The antifungal activity of YP TET, YP PRO and GLP PRO samples was evaluated using an azole-sensitive *C. albicans* strain (wild-type, WT SC5134), and the two azole-resistant strains ATCC 11651 and White 1. The strain White 1 is a fluconazole-resistant clinical isolate from Professor Theodore White’s laboratory (University of Missouri Kansas City) [27]. The antifungal activity was evaluated using a modified published microplate assay procedure previously described for the antimicrobial activity of YP-encapsulated terpenes [18,28]. The minimum inhibitory concentration (MIC) was determined as the concentration of fungicide that inhibits fungal growth, as measured by absorbance (650 nm), by more than 75%.

### 2.8. Checkerboard Microplate Assays

To characterize and quantify the antifungal activity of PRO, GET and the drug combinations over a range of concentrations, these compounds (unencapsulated or encapsulated in different YPs) were tested in a checkerboard 96-well plate format. To perform the assay, 100 µL of YPD was added to each well, followed by 100 µL of PRO or YP PRO to Rows A-H of column 1. Next, 1:1 serial dilution were performed from column 1 down to column 11, finally removing 100 µL from column 11. The second drug (GET or YP GET) was added to Row A of columns 1–12, and 1:1 serial dilution were performed from Row A down to Row G, finally removing 100 µL from Row G. Diluted *C. albicans* cells (100 µL, 10^6^ cells/mL) were added to all wells of the plate except H12. Initial (t = 0) and final (t = 18 h, 37 °C) absorbance readings were taken at 650 nm. The minimum inhibitory concentration was determined as the concentration that inhibits fungal growth as measured by absorbance by more than 75%. Heat maps of growth inhibition were generated using GraphPrism v 9.0. Contour plots (2D) and synergy calculations using zero interaction potency (ZIP) synergy scoring were generated using the SynergyFinder R online tool (https://synergyfinder.org, accessed on 14 March 2024) [29,30].

### 2.9. Statistical Analysis

All experiments were conducted with a minimum of three replicates and the reported data correspond to average values with standard deviation. For all two-group comparisons, a two-tailed Student’s test was used.

## 3. Results

### 3.1. Yeast Particle Encapsulation of Azoles

**Figure 1:**
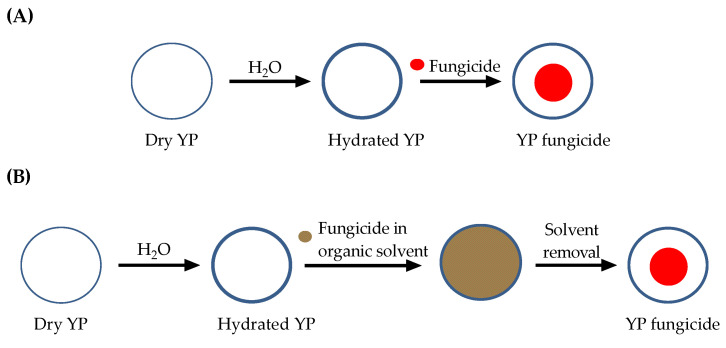
Schematics of two methods for loading of fungicides in yeast particles: (**A**) solvent-free loading method and (**B**) loading of payload in an organic solvent.

Yeast particles (YPs) are hollow, porous microparticles (3–5 µm) derived from Baker’s yeast [31]. The porous cell wall structure makes these particles excellent absorbent materials. We have previously demonstrated the high loading capacity of terpene encapsulation inside the hydrophobic cavity of YPs prepared by the passive diffusion of terpene through the porous cell walls of YPs [18,19]. Here, two loading methods were used for the encapsulation of azole fungicides, and the selection of the method for each payload was based on the physical state of the payload at room temperature (see Table 1 for chemical properties relevant to the selection of loading method in YPs). The first method (Figure 1A) is the solvent-free method previously used for encapsulation of terpenes [18,19]. This approach requires only a minimum volume of water to hydrate the pores of YPs, and then a payload with a high octanol/water partition coefficient (log P) that is a liquid or oil at room temperature (e.g., TET) is loaded by passive diffusion into the hydrophobic cavity of the particles. This is a very efficient method that does not require the use of organic solvents. The second method (Figure 1B) requires hydration of the YPs in a minimum volume of water followed by absorption of a highly concentrated solution of the payload in an organic solvent (e.g., PRO in acetone at 400 mg/mL). A minor disadvantage of this second method is the need to include steps for solvent removal.

**Figure 2:**
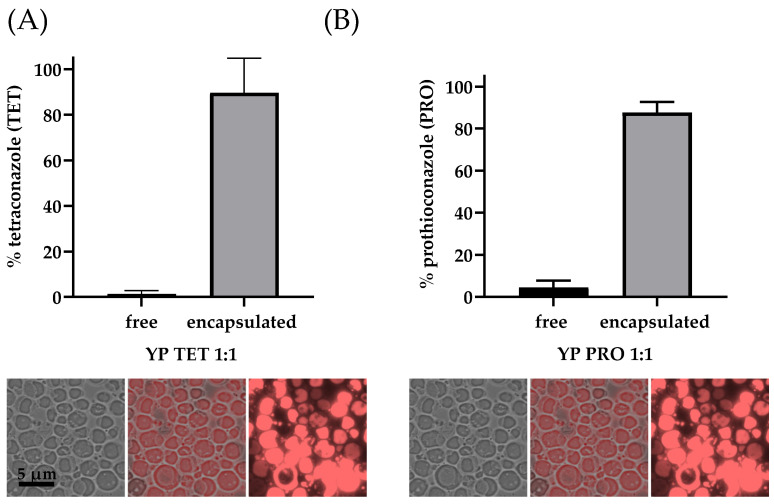
Encapsulation efficiency and microscopy images showing Nile-red-stained fungicides inside the cavity of YPs: (**A**) tetraconazole and (**B**) prothioconazole. Both compounds were loaded at a target weight ratio of 1:1 fungicide:YP.

Encapsulation of both fungicides was achieved with high efficiency, as determined quantitatively by HPLC analysis and qualitatively by microscopic Nile red staining, which allows for visualization of the payload loaded inside the hollow cavity of YPs (Figure 2). The solvent-free loading method was also used to prepare hyper-loaded TET samples similar to our previously reported hyper-loaded YP terpenes [18]. Samples with a 3:1 TET:YP ratio were produced with >90% TET encapsulation efficiency (Supplementary Materials).

**Figure 3:**
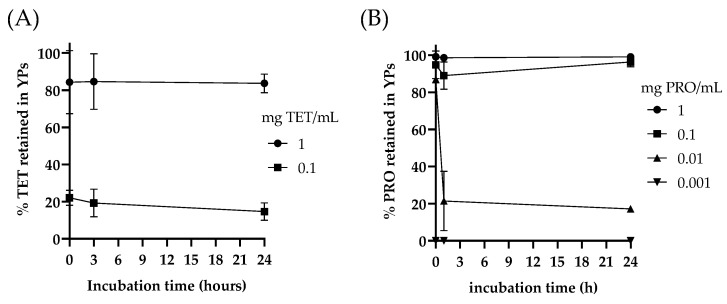
Kinetics of free and YP-encapsulated TET (Tetraconazole (**A**)) and PRO (prothioconazole (**B**)) released over time following dilution of YP fungicide samples in water at different fungicide concentrations and incubation at 23 °C.

The process of sustained fungicide release from YPs is based on diffusion of the payload of the particles and is a function of payload solubility in water. The encapsulation stability of the samples was evaluated by incubation of YP TET or YP PRO samples diluted at different fungicide concentrations in water. The results in Figure 3 show that YP TET remains stably encapsulated at 1 mg TET/mL (>80% TET inside YPs after 24, no burst release of TET in an emulsified form), but it is rapidly released upon dilution to 0.1 mg TET/mL (~20% TET inside YPs after <1 h). PRO is less water soluble than TET, and PRO remains stably encapsulated in YPs at 1 and 0.1 mg PRO/mL and releases the payload upon dilution at 0.01 mg PRO/mL.

### 3.2. Antifungal Activity of YP Azoles

**Figure 4:**
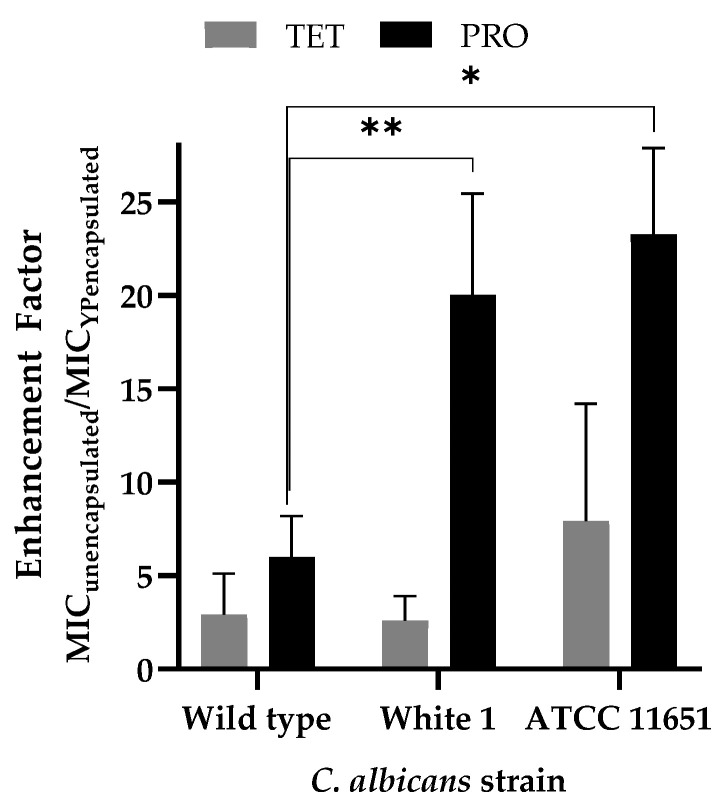
Effect of YP encapsulation on antifungal activity of azole fungicides reported as the ratio of the MIC 75% of unencapsulated and YP-encapsulated samples (statistically significant results were obtained between the paired samples, * *p* < 0.1, ** *p* < 0.05).

The antifungal activities of YP TET and YP PRO were evaluated in vitro against an azole-sensitive (wild-type) and two azole-resistant (White 1 and ATCC 11651) *C. albicans* strains. The results in Table 2 show that YP TET and YP PRO are more effective than the corresponding unencapsulated fungicide controls on both sensitive and azole-resistant strains. The ratio of active concentrations (MIC 75%) between the unencapsulated and encapsulated samples plotted in Figure 4 illustrates the enhanced effect of delivering the fungicides in YPs. Samples of YP TET were ~2.5 to 8 times more effective than unencapsulated TET (i.e., the MIC 75% of YP TET is ~2.5 to 8 times lower than MIC 75% of the unencapsulated TET) and YP PRO samples were six times more effective than unencapsulated PRO on sensitive *C. albicans* strain and 20–25 times more effective on azole-resistant strains.

The improvement of YP fungicides over unencapsulated fungicides on the azole-sensitive strain is likely due to the delivery of a more homogeneous YP fungicide suspension than the unencapsulated PRO powder suspension or TET oil mixture in YPD. This effect was previously observed for YP-encapsulated terpenes, with the encapsulated terpenes exhibiting a four-fold enhancement of antimicrobial activity compared to the unencapsulated compounds [18].

The effect of YP encapsulation on azole-resistant strains is significantly higher for PRO than for TET due to the strains not being highly resistant to unencapsulated TET. Further efforts of this investigation were focused on the YP PRO samples. Empty YPs were evaluated at the same YP concentrations as YP-PRO samples to confirm that the empty particles lack fungicidal activity. Empty particles admixed with unencapsulated PRO were also evaluated to determine if the enhanced effect on azole-strains can be obtained without the need for PRO encapsulation in YPs. The admixed samples have similar MIC 75% values as unencapsulated PRO for wild-type and White 1 strains and are only 2.5 times more active against the ATCC 11651 strain.

We hypothesized that the enhanced activity of YP PRO on azole-resistant strains was due to the presence of saponifiable lipids in YPs that could improve solubilization of PRO and uptake into the target pathogen. Improvement of physicochemical properties, such as solubility of fungicides to increase interaction with pathogens is critical to the development of novel fungicide formulations [33]. To confirm this hypothesis, PRO was encapsulated in glucan lipid particles (GLPs). GLPs are a highly purified form of YPs that retain non-saponifiable lipids necessary for the loading of hydrophobic payloads into the hollow cavity of the particles but lack the saponifiable lipids of YPs that could improve solubilization and release of hydrophobic payloads. Samples of GLP PRO were prepared following the same procedure as with YPs, and encapsulation at a target 1:1 PRO:GLP ratio was achieved with >90% efficiency (Supplementary Materials).

**Figure 5:**
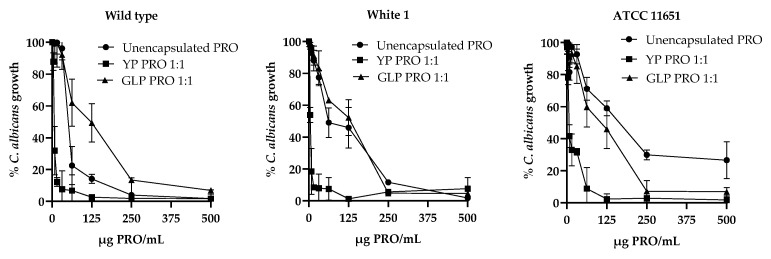
*Candida albicans* growth response curves with unencapsulated PRO and PRO encapsulated in YPs or GLPs.

The *C. albicans* growth response curves (Figure 5) with unencapsulated PRO and PRO encapsulated in YPs or GLPs show that encapsulation of PRO in GLPs inhibits PRO activity on the wild-type strain compared to unencapsulated PRO or YP PRO, and that GLP PRO has similar activity as unencapsulated PRO on azole-resistant/strains. These results support the hypothesis that the saponifiable lipid fraction present in YPs is largely responsible for enhancing the activity of PRO on azole-resistant strains.

The effect of YP PRO on azole-resistant strains requires further investigation on agricultural fungal pathogens. The use of YPs is suitable for agricultural applications but is not acceptable for parenteral pharmaceutical applications. The GLPs are suitable for pharmaceutical development, but it will be necessary to improve the activity of GLP fungicide samples by co-encapsulation with a sustained release agent to maximize formulation effectiveness.

We next focused on evaluating the possible synergy on azole-resistant strains by co-encapsulation of PRO and terpenes in YPs and GLPs.

### 3.3. Evaluation of Synergy of Prothioconazole and Terpenes Encapsulated in Yeast Particles and Glucan Lipid Particles against Azole-Resistant C. albicans

As previously published, the encapsulation of mixtures of the terpenes geraniol, eugenol and thymol (GET 2:1:2 weigh ratio) in YPs has been successfully implemented to develop and commercialize a fungicide product and mixtures of the terpenes geraniol and thymol as a nematicide product for agricultural applications [19,34]. A potential benefit of terpenes is that, given their generalized and multiple mechanisms of activity that evolved over millennia as plant defense molecules, terpenes are extremely difficult to make resistant to target pathogens. The combination of terpenes with antifungals (e.g., fluconazole) has been shown to act synergistically to overcome antifungal resistance [11,13,14].

**Figure 6:**
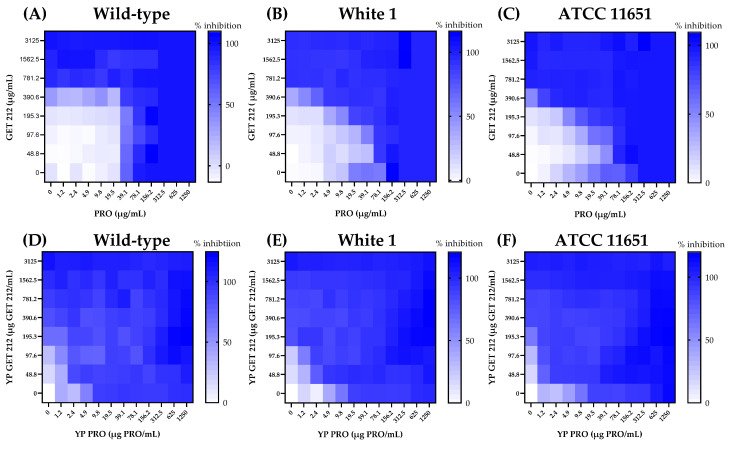
Heat maps showing effect of unencapsulated GET 212 and unencapsulated PRO (heat maps (**A**–**C**)), and YP GET 212 and YP PRO (heat maps (**D**–**F**)) on growth inhibition of sensitive and azole-resistant *C. albicans* strains.

The synergistic effects of PRO and GET212 were evaluated by conducting checkerboard MIC assays with unencapsulated and YP-encapsulated samples on the *C. albicans* strains. The heat maps in Figure 6 illustrate the inhibition activity of the different PRO and GET212 concentrations evaluated in the checkerboard assay. The results indicate that the YP PRO and YP GET212 samples are more potent (i.e., high % inhibition at low doses) than unencapsulated samples on the three *Candida* strains.

**Figure 7:**
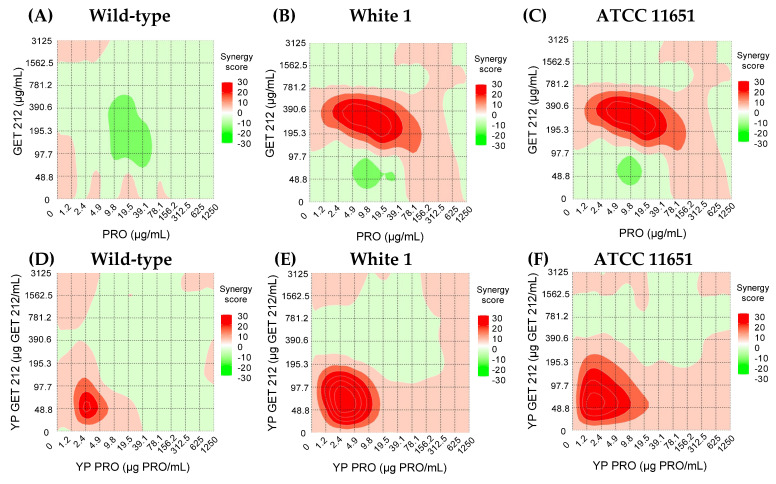
Two-dimensional contour plots showing synergy scores of unencapsulated GET212 and PRO (top (**A**–**C**)), and YP GET212 and YP PRO (bottom (**D**–**F**)). ZIP synergy scoring and 2D contour plots were generated using SynergyFinder R online tool (https://synergyfinder.org, accessed on 14 March 2024).

To assess if there is a synergistic effect between PRO and GET, the inhibition response from checkerboard assays was analyzed by ZIP synergy scoring generated using the SynergyFinder R online tool. The ZIP model captures interactions between two drugs by comparing the change in the potency of the inhibition response curves between the individual drugs and their combinations. The 2D contour plots in Figure 7 show the ZIP scoring values for the different free drug and YP-encapsulated drug checkerboard combinations on the three *C. albicans* strains. The interaction between the drugs is antagonistic for ZIP scores < −10, additive for score values between −10 and +10, and synergistic for ZIP scores > +10. The analysis of these 2D contour plots indicates that the unencapsulated PRO and GET212 primarily have an additive effect on wild-type *C. albicans*, and there is a concentration range that shows synergy in both azole-resistant strains (2.4 to 39.9 µg PRO/mL and 195.3 to 500 µg GET212/mL). The synergistic concentration range is at lower concentrations of PRO (1.2 to 9.8 µg/mL) and GET212 (24.4 to 146.5 µg/mL) when encapsulated in YPs, and the encapsulated samples show synergy on all three *Candida* strains. The improved synergy effect of YP-encapsulated samples compared to unencapsulated compounds is due to the production of a more homogenous YP drug suspension. The effect of YP encapsulation was also observed in the fungicidal results on wild-type *C. albicans* (Table 2) and previous work on the development of hyper-loaded YP terpenes [18]. The optimization of unencapsulated fungicide and terpenes will require a completely different formulation approach to identify proper ratios, solvent and surfactant conditions to produce a stable emulsion-based delivery system.

Next, we proceeded to prepare samples co-encapsulating PRO and GET212 at a ratio of 20:1 GET212:PRO in both YP and GLPs. The selected ratio corresponds to the ratio of the maximum YP PRO and YP GET212 concentrations showing synergy in the 2D contour analysis shown in Figure 7.

**Figure 8:**
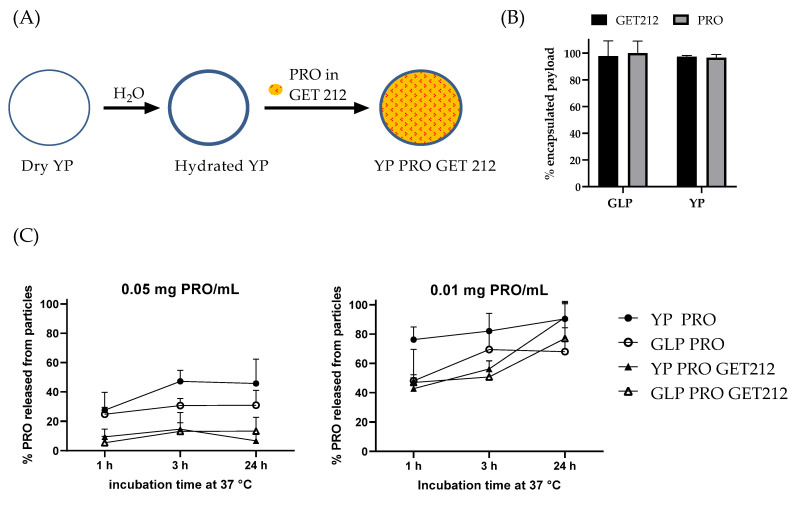
(**A**) Schematics of GET 212 and PRO co-encapsulation in YPs and GLPs, (**B**) GET212 and PRO encapsulation efficiency in YPs and GLPs in samples prepared at 0.055:1.1:1 PRO:GET 212:YP or GLP weight ratio, and (**C**) PRO release in water at 37 °C from control YP and GLP particles containing only PRO and samples co-encapsulating PRO and GET; samples were diluted at 0.05 mg PRO/mL (~2.3× higher concentration than maximum solubility of PRO in water) and at 0.01 mg PRO/mL (~2.2× lower concentration than maximum solubility of PRO in water).

YP and GLP samples co-encapsulating PRO and GET212 were prepared using GET212 as a loading solvent for PRO (Figure 8A) at a target weight ratio of 0.055:1.1:1 PRO:GET212:YP or GLP. The ratio of GET212:YP or GLP is the same that we have previously used to prepare YP GET212 for the development of a fungicide product for agricultural applications [18,19]. The ratio of PRO:GET212 of 1:20 is based on the synergy calculations results described above. Both payloads were efficiently encapsulated in GLPs and YPs (Figure 8B). YP and GLP samples encapsulating PRO alone or in combination with GET212 were evaluated for kinetics of PRO release. Unexpectedly, the results in Figure 8C show that the addition of GET212 slows the release of PRO from both YP and GLP, even at a concentration 2× below the maximum solubility of PRO in water.

**Figure 9:**
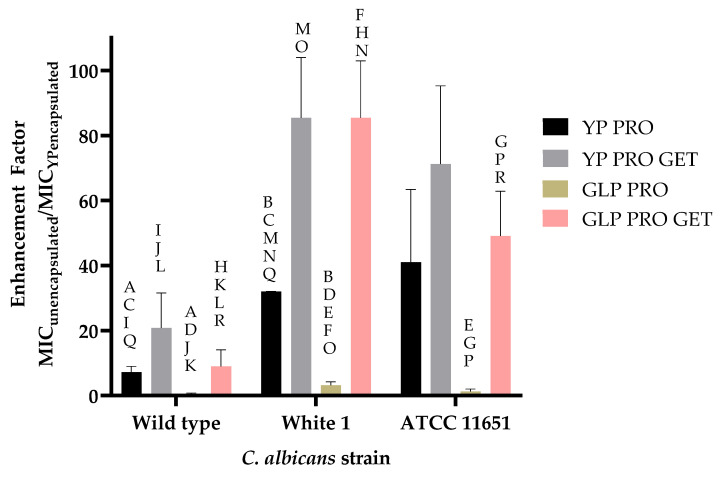
Effect of YP or GLP encapsulation on activity of prothioconazole, reported as the ratio of the MIC 75% of unencapsulated PRO and YP- or GLP-encapsulated PRO samples. Statistically significant results were obtained between the paired samples indicated with letter superscripts (A–E *p* < 0.005, F–H *p* < 0.01, I–R *p* < 0.05).

The samples co-encapsulating PRO and GET212 were evaluated for antifungal activity in microdilution assays. The results in Table 3 show that YP PRO and YP PRO GET212 have similar MIC 75% for PRO on sensitive and azole-resistant strains, but the activity of YP PRO GET212 is achieved with samples encapsulating <5% *w*/*w* PRO compared to the 50% *w*/*w* in YP PRO samples. Further, GLP PRO samples (1:1 PRO:GLP ratio) require high PRO concentrations on all strains, but samples co-encapsulating PRO with GET212 show improvement of PRO activity on sensitive and azole-resistant strains with MIC 75% values, similar to those of YP PRO and YP PRO GET. The MIC 75% ratio of unencapsulated and encapsulated PRO plotted in Figure 9 clearly illustrates a 20–40-fold improvement of YP encapsulation over unencapsulated PRO on azole-resistant strains and YP or GLP encapsulation in combination with terpenes (50–85-fold improvement). Although the results in Figure 7C indicate that GET212 does not improve solubilization of PRO, it is possible that GET212 released from the particles permeabilizes the *C. albicans* cell membrane or wall, allowing for improved uptake of PRO.

## 4. Discussion

Yeast particles offer several advantages as delivery vehicles, such as high payload loading capacity in the hollow cavity of the particles, payload protection from environmental stresses, biocompatibility, biodegradability, and possibility of controlled payload release. Additionally, highly purified forms of YPs developed for pharmaceutical applications provide for receptor-mediated targeted delivery to macrophages and dendritic via interaction of the of β-1,3-d glucan on the particle surface with cellular glucan receptors.

The encapsulation of hydrophobic payloads in YPs occurs by passive diffusion through the YP shell into its hydrophobic interior. This has been previously demonstrated for the efficient encapsulation of terpenes. Here, we show results of high payload loading capacity following the same solvent-free loading method as terpenes for the fungicide tetraconazole and the loading of prothioconazole using a suitable organic solvent for loading in YPs by a diffusion method. The YP TET and YP PRO samples prepared at 1:1 PRO or TET:YP weight ratios can be used to generate stable YP suspensions at 150 g YP/L of YP fungicide (15% *w*/*v*). These loading levels are higher than typical concentrations of TET in commercial products (e.g., 11.6% TET in Eminent 125 from Isagro [35]). Commercial PRO products like 4LSelect™ from Albaugh or Proline from Bayer Crop contain 41% TET *w*/*v* [36]. Although the 15% PRO in a formulation at 150 g YP/L is lower than commercial products, the preparation of YP PRO formulations has the advantages of eliminating the use of surfactants, the potential targeting of azole-resistant strains and reduction in PRO dosage by co-encapsulation with compounds like terpenes that exhibit a synergistic effect with PRO.

The release of fungicide from YP is based on diffusion out of the particles and is a function of the payload solubility in water. Both TET and PRO remain stably encapsulated in YP fungicide aqueous suspensions above the maximum solubility of the fungicide in water and release upon dilution below the maximum solubility of the fungicide in water (Figure 3). This encapsulation stability and diffusion-based release process is similar to the previously developed and commercialized YP terpene formulations for agricultural applications and is the basis for sustained active release.

The encapsulated TET and PRO samples exhibit slightly better activity than unencapsulated TET or PRO on azole-sensitive *C. albicans* and 5–20-fold better activity on azole-resistant strains (Table 2, Figure 4). The reduction in MIC for both YP TET and YP PRO on the azole-sensitive strain is likely due to delivering a more homogeneous fungicide suspension in YPs than the unencapsulated samples. The effect on azole-resistant strains was significantly higher (>10-fold enhancement) for PRO samples than TET, as none of the *C. albicans* strains used in this study showed strong resistance to unencapsulated TET, and further work evaluating the effect of YP encapsulation was focused on PRO samples. The enhanced activity of PRO in YP PRO samples is likely due to the interaction of PRO with saponifiable lipids in YPs that could improve PRO release from YPs and uptake into the *C. albicans* strains. The effect of the saponifiable lipids in YPs was confirmed by encapsulation of PRO in GLPs, a highly purified form of YPs that do not contain these saponifiable lipids. The growth inhibition dose response curves (Figure 5) show reduced activity of GLP PRO on both sensitive and azole-resistant strains compared to unencapsulated PRO or YP PRO samples.

The use of GLPs is critical for encapsulation of hydrophobic payloads for pharmaceutical applications. To trigger PRO release from GLPs and potentially target PRO to azole-resistant strains, we evaluated the combination of terpenes (GET212) with PRO. There are several advantages of using terpenes as chemosensitizers for antifungal application, such as (1) terpenes being natural compounds, (2) possessing antifungal activity but not at low enough concentrations like antifungal drugs, and (3) the main functions of terpenes being to permeabilize the cell wall and disrupt the fungal stress response, which can result in an additive or synergistic interaction with the primary antifungal drug [12]. Checkerboard assays of unencapsulated PRO and GET212 or YP-encapsulated drugs indicate that there is a synergistic effect between PRO and the GET212 terpene mixture at weight ratios from 10:1 to 20:1 GET212:PRO. Samples co-encapsulating GET212 and PRO at a 20:1 ratio in YPs or GLPs are active on sensitive and azole-resistant strains. The combination of GET212 and PRO offers the advantages of (1) ~10-fold reduction in the PRO weight content required for encapsulation (50% in YP PRO, 5.5% in YP or GLP PRO GET) and (2) overcomes the limitation of GLP PRO not being active due to the lack of saponifiable lipids, as the GET212 mixture improves PRO activity. The release kinetics results (Figure 8C) show that GET212 inhibited PRO release from YP or GLP PRO GET212 compared to YP or GLP PRO. These results do not correlate with the improved activity of YP and GLP PRO GET on *C. albicans*. It was expected that GET would improve PRO release from the particles and uptake by the target strains. However, it is possible that both payloads do not have to release from particles simultaneously to show synergy—a two-step mechanism could be taking place with (1) GET releasing faster from particles and disrupting *C. albicans* cell wall membrane and (2) PRO releasing slower and getting taken up by *C. albicans* due to a leaky membrane.

The methods developed to encapsulate PRO and TET in YPs or the combination of PRO and GET212 in YP and GLPs could be used for other synergistic antimicrobial hydrophobic drug combinations. The development of these materials is significant for the use of antimicrobial YP and GLP compositions for agricultural and pharmaceutical applications to combat the growing problem of drug-resistance.

## 5. Conclusions

Yeast particles can be used for the encapsulation of fungicides with high payload loading capacity, encapsulation efficiency and encapsulation stability. The presence of saponifiable lipids in YPs enhanced the activity of encapsulated PRO on azole-resistant *C. albicans* strains (YP PRO is >10-fold more active than unencapsulated PRO). The combination of PRO and terpenes (GET212) at a ratio of 20:1 GET212:PRO encapsulated in YPs or GLPs shows synergistic activity on both sensitive and azole-resistant *C. albicans* strains. These YP and GLP PRO GET212 combinations possess properties of interest for the development of YP and GLP materials for the targeting of fungal drug resistance in agricultural and pharmaceutical applications. The methods presented in this paper can be further investigated for other antimicrobial applications targeting drug resistance.

## 6. Patents

Hyperloaded Yeast Cell Wall Particle and Uses Thereof, G.R. Ostroff, E.R. Soto and F. Rus. US Patent App. 63/346,012. 26 May 2022.

## Figures and Tables

**Table 1:** Chemical structures of tetraconazole (TET) and prothioconazole (PRO) and their chemical properties relevant for the selection of loading method in YPs: TET can be loaded following a solvent-free method, and PRO can be loaded in YPs using acetone as the loading solvent.

	Tetraconazole (TET)	Prothioconazole (PRO)
Chemical structure	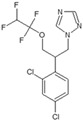	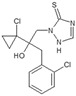
Log P	3.56	4.05
Melting point (°C)	6	139–144
Solubility in water	150 mg/L	22 mg/L
Solubility in acetone	-	440 mg/mL ^1^

^1^ All property values are from PubChem [32], except for the solubility of PRO in acetone (solubility value was determined in our laboratory).

**Table 2:** In vitro antifungal activity of YP PRO 1:1, unencapsulated (free) PRO suspension, unencapsulated PRO mixed with empty YPs and negative empty YP control on a susceptible (wild-type) and two azole-resistant *Candida albicans* strains. The MIC results represent the average of six replicate experiments. Statistically significant results were obtained between the paired samples indicated with letter superscripts (^A^ *p* < 0.005; ^B–E^ *p* < 0.01; ^F–J^ *p* < 0.05).

Sample	Minimum Inhibitory Concentration (MIC 75%) in µg PRO/mL on Wild-Type and Azole-Resistant *Candida albicans* Strains		
	Wild-Type	White 1	ATCC 11651
YP TET 1:1	10 ± 6 ^B^	18 ± 11	11 ± 6 ^F^
Unencapsulated TET	44 ± 17 ^B^	44 ± 26	60 ± 21 ^F^
YP PRO 1:1	12 ± 4 ^C,D^	19 ± 7 ^A,I,J^	12 ± 5 ^G^
Unencapsulated PRO	75 ± 28 ^C,H^	375 ± 161 ^A,E,J^	302 ± 108 ^G^
Unencapsulated PRO + empty YP	87 ± 34 ^D,E^	400 ± 137 ^A,E,J^	302 ± 118
Empty YP	Not active	Not active	Not active

**Table 3:** In vitro antifungal activity of unencapsulated (free) PRO suspension, particles encapsulating GET or PRO, and particles co-encapsulating both payloads at 0.055:1.1:1 PRO:GET212:particle weight ratio on a susceptible (wild-type) and two azole-resistant *C. albicans* strains. The MIC results represent the average of six replicate experiments. Statistically significant results were obtained between the paired samples indicated with letter superscripts (^A–J^ *p* < 0.005, ^K^ *p* < 0.01, ^L–P^ *p* < 0.05).

Sample	Dry Weight Composition			Minimum Inhibitory Concentration (MIC 75%) in µg/mL on Wild-Type and Azole-Resistant *Candida albicans* Strains					
	%PRO	%GET	%YP	Wild-Type		White 1		ATCC 11651	
				PRO	GET	PRO	GET	PRO	GET
Free PRO	100	-	-	78 ± 38 ^L,M,N^	-	500 ± 2 ^B,C,D,E^	-	417 ± 129 ^G^	-
YP GET	-	52.4	47.6	-	330 ± 16	-	327 ± 17	-	327 ± 17
YP PRO 1:1	50	-	50	11 ± 4 ^A,M^	-	16 ± 1 ^A,D,K^	-	10 ± 5 ^H^	-
YP GET PRO	2.6	51	47.4	3.9 ± 0.1 ^L^	82 ± 4	6 ± 2 ^B^	121 ± 41	6 ± 2 ^G,I,P^	121 ± 39
GLP PRO	50	-	50	200 ± 68 ^A,N,O^	-	175 ± 68 ^E,F,K^	-	333 ± 12 ^H,I,J,P^	-
GLP PRO GET	2.6	51	47.4	8 ± 1 ^O^	164 ± 8	6 ± 2 ^C,F^	121 ± 39	8 ± 6 ^J^	173 ± 112

## Data Availability

The original contributions presented in the study are included in the article/Supplementary Materials, further inquiries can be directed to the corresponding authors.

## Supplementary Materials

The following supporting information can be downloaded at: https://www.mdpi.com/article/10.3390/jfb15080203/s1, Figure S1: En-capsulation efficiency of tetraconazole (TET) encapsulated in YPs at a target weight ratio of 3:1 TET:YP; Figure S2: Encapsulation efficiency of prothioconazole (PRO) encapsulated in GLPs at a target weight ratio of 1:1 PRO:YP.
