# Supplementary material for: Yeast Particle Encapsulation of Azole Fungicides for Enhanced Treatment of Azole-Resistant Candida albicans
Source: J Funct Biomater. 2024 Jul 23;15(8):203. doi: 10.3390/jfb15080203 (PMC11355591; doi:10.3390/jfb15080203)
Supplement: Supplementary file 1 [file jfb-15-00203-s001.zip › jfb-3074999-supplementary.pdf]

Journal of *Functional Biomaterials*

MDPI

Article

Yeast Particle Encapsulation of Azole Fungicides for Enhanced Treatment of Azole-Resistant *Candida albicans*

Ernesto R. Soto, Florentina Rus and Gary R. Ostroff *

Program in Molecular Medicine, University of Massachusetts Medical School, Worcester, MA 01605, USA; ernesto.soto-villatoro@umassmed.edu (E.R.S.), florentina.rus@umassmed.edu (F.R.)
* Correspondence: gary.ostroff@umassmed.edu

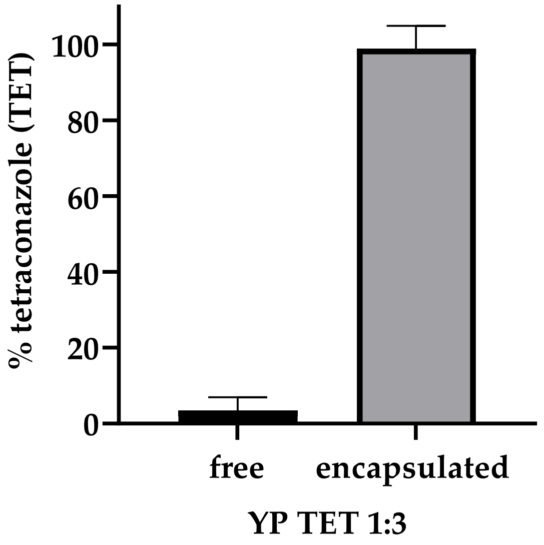

Figure S1. Encapsulation efficiency of tetraconazole (TET) encapsulated in YPs at a target weight ratio of 3:1 TET:YP.

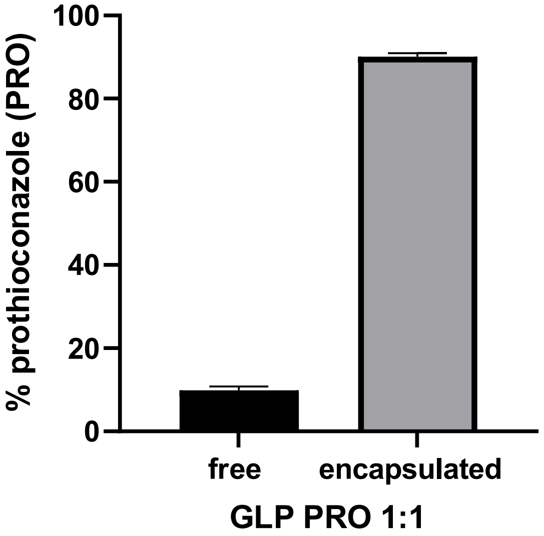

Figure S2. Encapsulation efficiency of prothioconazole (PRO) encapsulated in GLPs at a target weight ratio of 1:1 PRO:YP.

Citation: Soto, E.R.; Rus, F.; Ostroff, G.R. Yeast Particle Encapsulation of Azole Fungicides for Enhanced Treatment of Azole-Resistant *Candida albicans*. *J. Funct. Biomater.* **2024**, *15*, x. https://doi.org/10.3390/xxxxx

Academic Editor: Pankaj Vadgama

Received: 9 June 2024
Revised: 17 July 2024
Accepted: 18 July 2024
Published: 23 July 2024

Copyright: © 2024 by the authors. Submitted for possible open access publication under the terms and conditions of the Creative Commons Attribution (CC BY) license (https://creativecommons.org/licenses/by/4.0/).
